# Beyond the Holodomor: Current hunger in Ukraine and global food insecurity

**DOI:** 10.1002/puh2.149

**Published:** 2024-01-10

**Authors:** Nsikakabasi Samuel George, Faith Ohunene Okeji, Lucky Iseghehi

**Affiliations:** ^1^ School of Medicine and Population Health The University of Sheffield Sheffield UK; ^2^ École des hautes études en santé publique Rennes France; ^3^ Institute of Public Health, Jagiellonian University Krakow Poland; ^4^ School of Health Education and Life Sciences, Birmingham City University Birmingham UK; ^5^ eHealth Africa Kano Nigeria

Dear Editor,

The Holodomor, a devastating man‐made famine that occurred in Soviet Ukraine from 1932 to 1933, remains a haunting chapter in history, reminding us of the tragic consequences of political decisions on innocent lives. The Holodomor, which translates to ‘death by hunger’, was the result of policies instituted by Joseph Stalin as part of the First Five‐Year Plan, notably the forced collectivisation of agriculture in the Soviet Union [1]. These policies were planned and carried out by the Soviet Union and Russia with the intention of wiping out ethnic Ukrainians, bringing about their subjugation and obliterating their culture as well as their desire to form their own independent state outside from the Soviet Union [1]. This was coupled with the rapid industrialisation of the Soviet Union, which resulted in the requisition of grain and food from peasants. This led to a devastating famine and food insecurity that claimed the lives of millions of people, especially Ukrainians. Fast‐forward to the present and Ukraine is currently facing challenges related to war and hunger [1]. The country has been embroiled in a conflict with Russia with an invasion which has resulted in thousands of deaths, widespread displacement and a significant strain on resources. The current war has furthermore put a strain on Ukraine's ability to produce and distribute food, making it reliant on international aid and assistance and spotlighting the importance of national food security [2].

The issue of food insecurity, however, extends beyond the shores of Ukraine and continues to exist despite significant progress in its mitigation efforts like the UN and Turkiye mediated black sea grain deal in 2022 (currently suspended). In 2021, Russia was the second‐largest exporter of wheat, accounting for 15% of global exports, and Ukraine accounted for 10% of global wheat exports (Figure 1). However, since the war, trade disruptions, international sanctions and the displacement of farmers have disrupted agricultural production and supply chains. The 2023 Global Report on Food Crises reports that the enormous task of attaining the UN Sustainable Development Goal – Goal 2 of eradicating hunger by 2030 especially targets regarding food security, sustainable food production systems and addressing malnutrition is becoming increasingly arduous, given the persistent rise in the population experiencing elevated levels of acute food insecurity for the fourth consecutive year [3].

**FIGURE 1:**
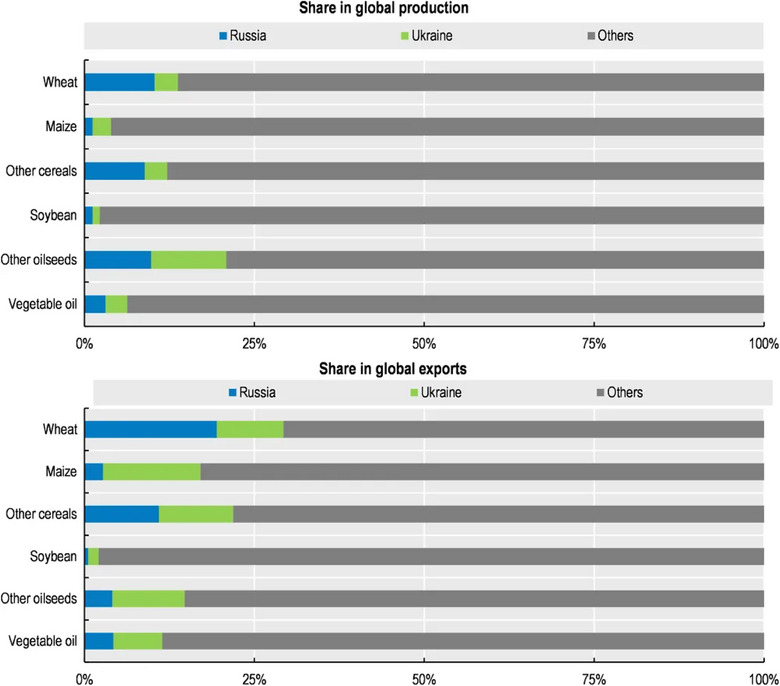
Shares of Russia and Ukraine in global production and exports of selected crops, 2016/17–2020/21 [4].

The Russia–Ukraine war has had profound health implications on the Russian–Ukrainian population and global dependent market with significant repercussions on the nutritional status of households [5]. The crisis has reduced access to nutritious food for millions of individuals and families. At the end of July 2023, an estimated eight million people in Ukraine faced insufficient food consumption [6]. This by far disproportionately affects families with low socioeconomic status, disposing children of these households to malnutrition and further increased susceptibility to diseases and health events [5]. The low access to nutritious food also causes a shift towards the consumption of less nutritious and highly processed foods which are risk factors for noncommunicable diseases like diabetes, obesity and cardiovascular diseases [7]. The war has also imposed additional costs on organisations like the World Food Programme (WFP) providing food humanitarian assistance to conflict‐affected and economically impoverished countries such as Yemen, Sudan and Lebanon [8]. Focusing on a nation, over 12% of Yemen's cereal supply comes from humanitarian food assistance, and the WFP strives to provide humanitarian aid to more than 13 million people which accounts for about 43% of Yemen's population [9]. The food supply was principally sourced from Ukraine and Russia, and thus, the current crises have great implications on the health and well‐being of the populace.

Global food insecurity is a multifaceted problem that requires coordinated efforts from governments, international organisations and civil society. These coordinated efforts involve not only health policies but also economic, social and political strategies. First, efforts through diplomacy should be made to resolve conflicts via peaceful means. These efforts encompass seeking peaceful and negotiated solutions to conflicts and providing economic aid to nations grappling with widespread and acute hunger, among other initiatives like the Black Sea Grain Initiative. Second, sustainable agriculture must be supported. Investing in sustainable agricultural practices can enhance food production while minimising environmental degradation. Implementing resilient farming techniques can help communities adapt to a changing climate. Considering that the reliance on chemical fertilisers is unsustainable due to current costs, countries should consider using biofertilisers as an intervention to mitigate the looming surge of food insecurity. Biofertilisers have proven to enhance food production and are considered safer for consumers, making them a preferable option for cultivating safer crops and addressing global food security [10]. Third, food distribution must be improved. Creating efficient and equitable food distribution systems can help ensure that food reaches those who need it most. Reducing food waste throughout the supply chain is also crucial. Additionally, ending trade and export restrictions will enable the free circulation of food produced. Fourth, there is a need to strengthen social protection measures and safety nets. Establishing robust social safety nets can provide a buffer against food insecurity during times of crisis, ensuring that vulnerable populations have access to adequate nutrition and essential services. Fifth, building resilient communities that can withstand shocks, such as conflicts or climate‐related disasters, is vital. Policies for health such as maternal and child health, education, health systems strengthening and ensuring healthcare access can exacerbate food insecurity and malnutrition. Health education here will enable individuals and families to make health choices on food consumption and utilise available food sources in combinations that are health‐seeking and reduce risk to noncommunicable diseases and other adverse health conditions.

Revisiting the Holodomor in the context of the current war and hunger in Ukraine reminds us of the devastating consequences of political decisions and conflicts on food security. The parallels between these tragedies emphasise the urgency of addressing global food insecurity, a complex issue with far‐reaching consequences. By taking a decisive stand to first prioritise human rights, prioritise conflict prevention and resolution, foster peace, invest in sustainable agriculture and climate resilience, improve food distribution, health systems strengthening, strengthen social safety nets and enhance resilience, the world can move towards a future where food security is a universal reality.

## AUTHOR CONTRIBUTIONS

Nsikakabasi Samuel George performed the conceptualisation and design of the study. Nsikakabasi Samuel George, Faith Ohunene Okeji and Lucky Iseghehi contributed to the acquisition and interpretation of data for the study. Nsikakabasi Samuel George critically reviewed the draft and produced the final manuscript. Nsikakabasi Samuel George, Faith Ohunene Okeji and Lucky Iseghehi read and approved the final manuscript.

## CONFLICT OF INTEREST STATEMENT

The authors declare no conflicts of interest.

## FUNDING INFORMATION

This research did not receive any specific grant from funding agencies in the public, commercial or not‐for‐profit sectors.

## Data Availability

The authors confirm that the data supporting the findings of this study are available within the article.

## References

[1] Bezo B , Maggi S . Living in “survival mode”: intergenerational transmission of trauma from the Holodomor genocide of 1932–1933 in Ukraine. Soc Sci Med. 2015;134:87‐94. 10.1016/j.socscimed.2015.04.009 25931287

[2] Ben Hassen T , El Bilali H . Impacts of the Russia‐Ukraine war on global food security: towards more sustainable and resilient food systems? Foods. 2022;11(15):2301. 10.3390/foods11152301 35954068 PMC9368568

[3] FSIN and Global Network Against Food Crises . 2023 Global Report on Food Crises: Joint Analysis for Better Decisions . 2023. https://www.fsinplatform.org/sites/default/files/resources/files/GRFC2023‐hi‐res.pdf

[4] OECD/FAO . OECD‐FAO Agricultural Outlook (Edition 2022) . OECD Agriculture Statistics (database); 2022. 10.1787/13d66b76-en

[5] Yazbeck N , Mansour R , Salame H , Chahine NB , Hoteit M . The Ukraine–Russia war is deepening food insecurity, unhealthy dietary patterns and the lack of dietary diversity in Lebanon: prevalence, correlates and findings from a national cross‐sectional study. Nutrients. 2022;14(17):3504‐3504. 10.3390/nu14173504 36079761 PMC9460330

[6] Statista . Number of People Facing Insufficient Food Consumption in Ukraine from May to July 2023 . 2023. Accessed October 16, 2023. https://www.statista.com/statistics/1312674/ukraine‐people‐with‐insufficient‐food‐consumption/

[7] Welsh C . Russia, Ukraine, and Global Food Security: A One‐Year Assessment . Center for Strategic & International Studies. 2023. Accessed October 15, 2023. https://www.csis.org/analysis/russia‐ukraine‐and‐global‐food‐security‐one‐year‐assessment

[8] Ahsan H , Alvi AS , Yaseen M . Russia‐Ukraine war: impacts on world food security. J Dev Soc Sci. 2023;4(2):676‐682. 10.47205/jdss.2023(4-II)59

[9] FAO . Yemen Country Brief: GIEWS . FAO; 2023. Accessed October 10, 2023. https://www.fao.org/giews/countrybrief/country.jsp?code=YEM&lang=ar

[10] Daniel AI , Fadaka AO , Gokul A , et al. Biofertilizer: the future of food security and food safety. Microorganisms. 2022;10(6):1220. 10.3390/microorganisms10061220 35744738 PMC9227430

